# Retraction: Genetic Variations in Plasma Circulating DNA of HBV-Related Hepatocellular Carcinoma Patients Predict Recurrence after Liver Transplantation

**DOI:** 10.1371/journal.pone.0222258

**Published:** 2019-09-04

**Authors:** The PLOS ONE Editors

Concerns have been raised that the transplants performed in the local context at the time of procedures reported in this article [1] may have involved organs/tissues procured from prisoners [2].

International ethics standards call for transparency in organ donor and transplantation programs and clear informed consent procedures including considerations to ensure that donors are not subject to coercion. It was noted in [1] that deceased donors provided organs for the transplant procedures in the reported study, but details as to the donor sources and methods of obtaining informed consent from donors were not reported. In response to journal inquiries the authors did not clarify these issues or the cause(s) of donor death, although they confirmed that the transplants were done in compliance with local laws and regulations in place at the time and provided documentation pertaining to ethical approval for the study.

The authors requested withdrawal of the article in response to the journal’s queries about the above issues, and the corresponding author raised concerns about the premise of the journal investigation.

Owing to insufficient reporting and unresolved concerns around the source of transplanted organs and whether they included organs from prisoners, and in compliance with international ethical standards for organ/tissue donation and transplantation, the *PLOS ONE* Editors retract this article.
